# Periodontitis and Chronic Liver Disease: Mechanistic Insights Focusing on *Porphyromonas gingivalis*—A Narrative Review

**DOI:** 10.3390/microorganisms14040736

**Published:** 2026-03-26

**Authors:** Yue Ying, Yuwei Nie, Jiahui Zhao, Qin Dong, Meixian Chen, Aijia Jiang, Nan Liu, Tong Xu, Junchao Liu, Yaping Pan, Li Lin, Dongmei Zhang

**Affiliations:** 1 Department of Periodontics, School of Stomatology, China Medical University, Shenyang 110002, China; 2023121967@cmu.edu.cn (Y.Y.); xxiuu162@163.com (Y.N.); 2023122032@cmu.edu.cn (J.Z.); dongqin@cmu.edu.cn (Q.D.); chenmeixian555@163.com (M.C.); 20172238@cmu.edu.cn (A.J.); 2023240171@cmu.edu.cn (N.L.); tongxuzi@163.com (T.X.); 2 Department of Periodontics and Oral Biology, School of Stomatology, China Medical University, Shenyang 110002, China; liujunchao1986@126.com (J.L.); yppan@cmu.edu.cn (Y.P.); linli_74k@163.com (L.L.)

**Keywords:** periodontitis, *Porphyromonas gingivalis*, chronic liver diseases, ferroptosis, immune evasion, endothelial–mesenchymal transition

## Abstract

*Porphyromonas gingivalis* (*P. gingivalis*), a keystone pathogen in periodontitis, has been increasingly implicated in compromising hepatic health and exacerbating the pathogenesis of liver diseases, including metabolic dysfunction-associated steatotic liver disease (MASLD), chronic hepatitis, and cirrhosis. Current studies have identified three well-established pathways through which periodontitis contributes to chronic liver disease progression: systemic inflammatory responses, liver cells dysfunction, and gut microbiota dysbiosis. This review systematically elucidates the associations between periodontitis and chronic liver disorders, consolidates evidence on the canonical molecular mechanisms involved, and further proposes potential yet understudied pathways such as ferroptosis, immune evasion, and endothelial–mesenchymal transition (EndMT). By integrating these insights, this work aims to provide novel perspectives for mitigating the systemic adverse effects of periodontitis while offering a theoretical foundation for future research and clinical therapeutic strategies.

## 1. Introduction

Periodontitis is a chronic inflammatory disease caused by mixed bacterial synergistic interactions, leading to gingival bleeding, oral malodor, alveolar bone resorption, and ultimately, tooth loosening and loss [1,2]. Periodontal pathogens primarily reside in non-attached subgingival plaque, most of which are opportunistic pathogens [3,4]. These pathogens do not exhibit pathogenic effects when in a balanced state with the host but become pathogenic when the equilibrium is disrupted or the host’s immunity is compromised [5].

Periodontitis has been increasingly linked to chronic liver diseases (CLDs) [6,7,8,9,10,11]. While *Porphyromonas gingivalis* (*P. gingivalis*) is the most studied key pathogen in this association, other periodontal bacteria such as *Tannerella forsythia* (*T. forsythia*) [9,10,11], *Fusobacterium nucleatum* (*F. nucleatum*) [9,12,13], and *Treponema denticola* (*T. denticola*) [9,10,11] also contribute. This review, therefore, selects *P. gingivalis* as the entry point to explore the underlying mechanisms of periodontitis, while briefly addressing the roles of other relevant bacteria.

*P. gingivalis* is a common Gram-negative anaerobic bacterium in the oral cavity and is recognized as one of the most critical periodontal pathogens [14]. Its multiple virulence factors can act individually or synergistically to promote bacterial colonization, activate inflammation, and initiate bone resorption, thereby destroying periodontal tissues and contributing to the development of various systemic diseases through these mechanisms [15,16] (Table 1).

Specifically, lipopolysaccharide (LPS) upregulates the receptor activator of nuclear factor kappa-B ligand (RANKL) expression, accelerating osteoclast differentiation and alveolar bone resorption [17]. It activates Toll-like receptor 2 (TLR2), leading to the production of pro-inflammatory cytokines such as tumor necrosis factor-α (TNF-α), interleukin-1β (IL-1β), and interleukin-6 (IL-6), thereby amplifying the local inflammatory response [18]. Additionally, it suppresses interleukin-12 (IL-12) production and interferes with antigen presentation, promoting immune evasion [19]. The capsule shields surface antigens, inhibiting complement activation and phagocytosis by immune cells, thereby mediating immune suppression [17,20]. It further enhances bacterial adhesion to biofilms (primarily harboring the red complex (*P. gingivalis*, *T. forsythia*, *T. denticola*), promoting colonization [21]. Similarly, fimbriae bind to β1 integrins (e.g., α5β1) on host epithelial cell surfaces, mediating bacterial adhesion, promoting polyspecies biofilm formation, and facilitating bacterial internalization and the invasion of epithelial cells [17,22]. They also promote osteoclast differentiation (via a similar mechanism to that of LPS) [23]. Furthermore, the outer membrane vesicles (OMVs) carry virulence factors such as LPS and gingipains, promoting adhesion and biofilm stability [17]. OMVs additionally target Chromobox 5 (which promotes and maintains the formation and stability of heterochromatin, leading to gene silencing) via sRNA45033 binding to its 3′ untranslated region, thereby inducing mitochondrial dysfunction and promoting periodontal ligament cell apoptosis [24]. Meanwhile, they activate the mitogen-activated protein kinase (MAPK) pathway, thereby releasing inflammatory cytokines to amplify the local inflammatory response [25].

**Table 1:** Virulence factors of *Porphyromonas gingivalis* and their pathogenic effects.

Virulence Factors	Pathogenic Effect	References
LPS	Accelerates bone resorption. Amplifies the local inflammatory response. Induces immune evasion.	[17,18,19]
Capsule	Mediates immune tolerance. Enhances bacterial adhesion colonization.	[17,20,21]
Fimbriae	Activate osteoclast differentiation. Mediate epithelial cell adhesion and promote invasion. Maintain biofilm stability.	[17,22,23]
OMVs	Promote bacterial adhesion and colonization. Induce periodontal ligament cell apoptosis. Amplify the local inflammatory response.	[17,24,25]
Gingipains	Promote the colonization of bacteria. Degrade host proteins to escape immune defense.	[17,26,27,28]
Collagenases	Decompose collagen fibers. Destroy periodontal tissue and gingival connective tissue.	[17,29,30]
Hmu hemoglobin utilization system	Degrades heme to release iron ions and provides nutrients for *P. gingivalis*. Supports bacterial growth and colonization. Enhances the synthesis of others virulence factors.	[17,31,32]

Abbreviations: LPS, lipopolysaccharide; OMVs, outer membrane vesicles; *P. gingivalis*, *Porphyromonas gingivalis*.

Gingipains promote the growth of *T. forsythia* and the aggregation of *T. denticola* in biofilms [17,26]; they degrade complement components C3/C5 and antimicrobial peptides, thereby disrupting immune defenses [27,28]. In addition, collagenases hydrolyze type I collagen, leading to the breakdown of collagen fibers and the connective tissue matrix, and activate matrix metalloproteinase-2 (MMP-2), matrix metalloproteinase-9 (MMP-9), and other matrix metalloproteinases (MMPs) to further destroy periodontal tissues. Notably, the Hmu hemoglobin utilization system degrades hemoglobin to release iron ions and heme, providing essential nutrients for bacterial growth [17,31]. It serves as a barrier against oxidative damage from H_2_O_2_ and other agents, promoting the synthesis and enhancing the activity of gingipains [31,32].

**Figure 1:**
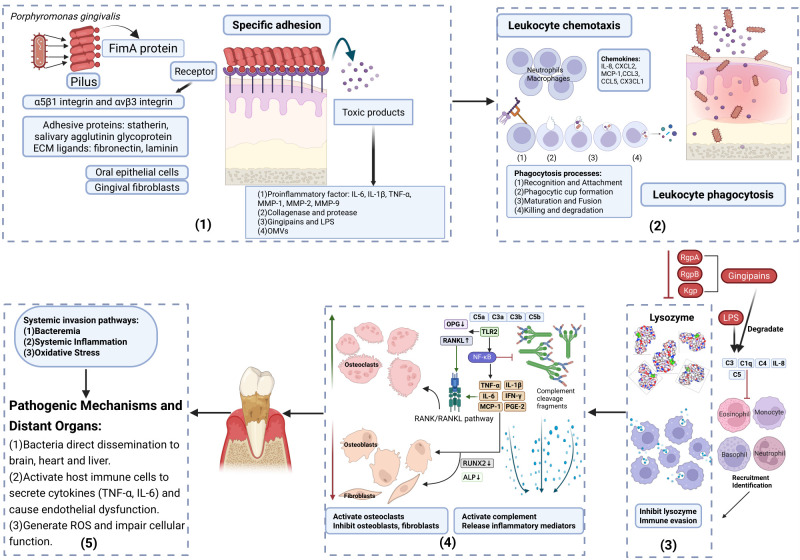
The local pathogenic mechanisms and systemic dissemination pathways of *Porphyromonas gingivalis*. Key pathogenic cascade: (1) Tissue-specific adhesion: FimA fimbriae bind to various host surface molecules, including adhesive proteins (statherin, salivary agglutinin glycoprotein), extracellular matrix ligands (fibronectin, laminin), and transmembrane receptors such as α5β1 integrin and αvβ3 integrin, leading to the release of pro-inflammatory factors IL-6, IL-1β, TNF-α, MMP-1, MMP-2, and MMP-9; this facilitates bacterial adhesion, invasion, and biofilm formation. (2) Dysregulation of immune cell chemotaxis and epithelial barrier disruption: Virulence factors (gingipains, LPS, collagenase) regulate chemokines (such as IL-8, CXCL2, MCP-1, CCL3, CCL5, and CX3CL1) through concentration-dependent cleavage or degradation, impairing phagocytic capacity of immune cells and disrupting epithelial tight junctions. (3) Immune evasion: Gingipains (RgpA, RgpB, Kgp) degrade and inactivate the lysozyme, reducing its ability to hydrolyze bacterial peptidoglycan and support opsonization. Simultaneously, they cleave key complement components (C1q, C3, C4, C5), preventing effective deposition and membrane attack complex formation. This dual subversion disrupts the synergistic promotion of phagocytosis by the lysozyme and complement, thereby suppressing immune cell recruitment, recognition, and bacterial clearance. (4) Chronic low-grade inflammation and osteoclastic response: Activation of the TLR2 pathway upregulates RANKL, downregulates OPG, RUNX2 and ALP, and synergizes with pro-inflammatory factors such as IL-6 to activate the RANK/RANKL pathway, promoting osteoclast differentiation while inhibiting osteoblast differentiation/mineralization and gingival fibroblast collagen synthesis; sustained release of inflammatory mediators occurs, including pro-inflammatory cytokines TNF-α, IL-1β, IL-6, and IFN-γ and chemokines IL-8, MCP-1, and PGE-2, creating a chronic low-grade inflammatory microenvironment. Upward arrows indicate upregulation, and downward arrows indicate downregulation. (5) Systemic induction of remote organ dysfunction: Bacteria disseminate via bacteremia to distant organs (liver, heart, and brain), triggering systemic inflammatory response and oxidative stress (ROS accumulation), thereby promoting progression of systemic diseases. Abbreviations: ECM, extracellular matrix; MMP-1, matrix metalloproteinase-1; IL-8, interleukin-8; MCP-1, monocyte chemoattractant protein 1; CCL3, C-C motif chemokine ligand 3; CX3CL1, C-X3-C motif chemokine ligand 1; RgpA, arginine-specific gingipain A; RgpB, arginine-specific gingipain B; Kgp, lysine-specific gingipain; OPG, osteoprotegerin; RANK, receptor activator of nuclear factor kappa-B; NF-κB, nuclear factor kappa-light-chain-enhancer of activated B cells; IFN-γ, interferon gamma; PGE-2, prostaglandin E_2_; RUNX2, runt-related transcription factor 2; ALP, alkaline phosphatase; ROS, reactive oxygen species; FimA, Fimbriae protein A; TNF-α,tumor necrosis factor-alpha; IL-1β, interleukin-1 beta; IL-6,interleukin-6; MMP-2,matrix metalloproteinase-2; MMP-9,matrix metalloproteinase-9; TLR2, Toll-like receptor 2; C5a, complement component 5a; C3a, complement component 3a; C3b, complement component 3b; C5b, complement component 5b; C3, complement component 3; C1q, complement component 1q; C4, complement component 4; C5, complement component 5; AS, atherosclerosis. Created with BioRender.com and based on [17,18,19,22,23,27,28,29,30,33,34,35,36,37,38].

As a key pathogenic bacterium, *P. gingivalis* induces local tissue damage and promotes systemic diseases through a series of mechanisms. Specifically, it relies on specific adhesion, mediated by FimA, to surface receptors (including statherin, salivary agglutinin glycoprotein, α5β1 integrin, fibronectin, and laminin) [17,22,23,33,34,35] and releases toxic products such as LPS, collagenase, and gingipains. These factors induce the dysregulation of immune cell chemotaxis, the release of chemokines (such as C-C motif chemokine ligand 5 (CCL5), C-X-C motif chemokine ligand 8 (CXCL8), and C-X-C motif chemokine ligand 2 (CXCL2)), and the disruption of the epithelial barrier [17,18,19,27,28,30]. By inhibiting immune cell recruitment and recognition, the bacterium achieves immune evasion, activates inflammatory signaling pathways, and triggers osteoclastic responses [17,29,30,36,37]. In addition, *P. gingivalis* induces remote organ dysfunction through various pathways [17,38] (Figure 1). These interconnected events collectively drive the pathogenicity of *P. gingivalis*.

Periodontitis has been found to be closely associated with systemic diseases such as diabetes [39], atherosclerosis (AS) [40], and chronic kidney disease [41]. In recent years, research on the relationship between periodontitis and liver diseases has gradually attracted great attention. Previous studies have identified systemic inflammatory responses, impaired hepatocyte function, and oral–gut–liver axis dysregulation as core pathways by which periodontal pathogens induce liver injury [7,8,9,13,42,43].

The progression of liver diseases is multifactorial in nature, and the interactions between periodontitis and liver diseases are highly complex. Although there is increasing interest in the impact of periodontitis on liver diseases, existing research in this area remains limited and often yields inconclusive results. Immune evasion, ferroptosis, and EndMT are emerging topics in recent research; however, only a few studies have explored how periodontitis promotes liver disease progression by regulating these pathways [44,45,46,47,48,49,50,51,52,53]. Our review aims to provide an updated and comprehensive overview of the current understanding by synthesizing the existing literature, uncovering novel pathogenic mechanisms, and highlighting key areas for future research. By offering new insights into the interplay between these two conditions, this review hopes to foster interdisciplinary collaboration between dentistry and hepatology and improve patient outcomes.

## 2. Periodontitis and Liver Diseases

Emerging evidence highlights significant associations between periodontitis and chronic liver conditions, including metabolic dysfunction-associated steatotic liver disease (MASLD, the updated nomenclature, formerly known as non-alcoholic fatty liver disease (NAFLD)) [7,54,55,56,57,58,59,60,61,62,63], hepatitis [7,47,64,65,66,67,68,69,70], cirrhosis [7,63,71,72,73,74,75,76,77,78,79,80,81], and hepatocellular carcinoma (HCC) [7,71,82,83,84,85,86].

### 2.1. Periodontitis and Non-Alcoholic Fatty Liver Disease

MASLD is linked to metabolic dysfunction. Its diagnosis relies on cardiometabolic risk factors (including overweight, diabetes, hypertension, and dyslipidemia), while excluding other clear etiologies and excessive alcohol consumption (women < 20 g/d, men < 30 g/d) [87]. It has replaced the previous definition of the previous term NAFLD [54], but the diagnostic criterion requires the confirmation of ≥5% hepatocellular steatosis [87,88,89]. Subtypes include metabolic dysfunction-associated steatotic liver disease, metabolic dysfunction-associated steatohepatitis (MASH), and MASH-related fibrosis/cirrhosis [89]. Epidemiological data show that MASLD has become a major cause of cirrhosis globally [90] and that it is closely associated with metabolic abnormalities, including obesity [90], type 2 diabetes [91], insulin resistance [91], and metabolic syndrome [43].

In recent years, there has been a growing body of literature regarding the association between periodontitis and MASLD [7,55,56,57,58,59,60,61,62]. Furusho et al. [55] found that *P. gingivalis* infection promotes the pathological progression of high-fat diet-induced MASH, with bacterial load correlating to fibrosis severity. The type IV pili of highly tissue-invasive *P. gingivalis* were identified as an independent risk factor for advanced fibrosis in MASLD patients (odds ratio (OR) = 2.081), and periodontitis is more severe in MASLD patients than in systemically healthy individuals [56]. In contrast to previous findings [56], Yoneda et al. [57] reported that type II pili of *P. gingivalis*, which are prevalent in periodontitis plaque, exhibit a stronger association with MASH progression compared to that of type IV pili. This discrepancy may stem from differing sample sources, i.e., serum [56] versus saliva [57]. Another line of evidence comes from therapeutic experiments, e.g., azithromycin-mediated *P. gingivalis* clearance suppressed MASH progression [58], though limitations include unassessed serum aspartate aminotransferase (AST)/alanine aminotransferase (ALT) levels and unverified long-term efficacy post-treatment. Furthermore, the association between *F. nucleatum* and MASLD progression has also been confirmed in animal experiments [59]. Specifically, gingival sulcus injection of *F. nucleatum* suspension significantly increased hepatic collagen deposition in apolipoprotein E-deficient mice, accompanied by elevated serum ALT, AST, and lipid levels, synergistically promoting MASLD progression [59]. However, this effect is currently limited to experimental animal evidence and has not been verified by in vitro studies.

At present, more evidence has confirmed that there is a relatively obvious association between the two conditions [60,61]. Most of the literature tends to support that there is a significant association without causal hierarchy. Further research is required to verify this association.

### 2.2. Periodontitis and Chronic Hepatitis

Chronic hepatitis is characterized by persistent hepatic inflammation lasting more than six months and caused by a variety of factors, including viral infections (such as hepatitis B virus (HBV) [92] and hepatitis C virus (HCV) [93]), autoimmune conditions [94], alcohol consumption [95], and medications [96].

Chronic hepatitis B (CHB) remains a major chronic liver disease driver, showing association with hepatic steatosis [97,98]. HCV-induced chronic hepatitis (CHC), which is primarily transmitted through blood [99], significantly contributes to the development of HCC [100]. Autoimmune hepatitis (AIH), prevalent in Western populations, can progress to cirrhosis or liver failure [101]. Alcoholic steatohepatitis is a liver injury caused by long-term alcohol consumption, diagnosed through clinical history, hepatic steatosis, and immune infiltration [102].

Evidence underscores the association between periodontitis and chronic hepatitis [7,47,64,65,66,67,68,69,70], particularly viral hepatitis. The initial link between *P. gingivalis* and hepatitis stemmed from observations of higher HBV DNA levels in saliva than in serum [64]. Annemiek proposed that this discrepancy might indicate the salivary glands as one of the HBV replication sites [64], and saliva is also considered a potential transmission medium for HBV. If HBV can reach the liver via salivary pathways, periodontal pathogens in saliva may similarly be associated with hepatic tissue through comparable mechanisms. Furthermore, salivary interleukin-2 levels are significantly higher in periodontitis patients with concurrent HBV infection than in those without HBV [70]. Another key finding is that salivary occult blood positively correlates with HBV viral load, suggesting that improved periodontal status may reduce horizontal HBV transmission; however, this study only focused on remaining teeth count and lacked systematic clinical periodontal evaluations [65]. These findings further indicate an association between periodontitis and HBV infection. In periodontitis patients with CHC, gingival crevicular fluid cytokine levels were higher than in systemically healthy patients and positively correlated with periodontal clinical parameters [66], which was confirmed by another study [67]. A Japanese researcher found that elevated serum ALT levels were significantly associated with the number of sites with a probing depth ≥ 6 mm (OR = 1.10) and clinical attachment level (CAL) ≥ 6 mm (OR = 1.03) [68]. Analysis of the NHANES dataset also confirmed that hepatitis virus infection significantly increased periodontitis prevalence, and periodontitis severity was associated with higher rates of viral hepatitis infection [69]. Animal experiments provided supporting evidence, e.g., the administration of *P. gingivalis* in alcohol-related liver disease (ALD) mice elevated AST levels and exacerbated alcohol-induced liver dysfunction [47]. However, conflicting findings exist; Tsai reported that periodontitis was significantly associated with liver inflammation only in the presence of systemic inflammation [103].

Generally, the connection between periodontitis and chronic hepatitis is not as clear as that with MASLD; most evidence has established the association between viral hepatitis and periodontitis, while evidence regarding non-viral chronic hepatitis (such as AIH) is scarce; thus, more accurate and direct evidence is still required.

### 2.3. Periodontitis and Cirrhosis

Cirrhosis is a chronic liver disease that results from progressive fibrosis and nodular regeneration, leading to architectural distortion and parenchymal hardening [104]. Etiologies include MASLD, CHB, and CHC [105].

Costa found that the prevalence of periodontitis was significantly higher in patients with liver cirrhosis than in the control group [72]. Another study reported more severe alveolar bone loss and periodontal destruction in patients with alcoholic liver cirrhosis than in systemically healthy individuals [80], suggesting that liver cirrhosis may be a risk factor for periodontitis [72,80]. Other clinical studies also confirmed that periodontitis accelerates the progression of alcoholic liver cirrhosis (shortening the time from diagnosis to the need for liver transplantation) [63,73]. However, there is currently no definitive evidence to establish a clear causal relationship.

In addition, the abundance of red complex bacteria (*P. gingivalis*, *T. forsythia*, and *T. denticola*) in the subgingival microbiota of periodontitis patients with cirrhosis was significantly lower than in those with conventional periodontitis [74], which might be attributable to cirrhosis-related immune alterations that shift bacterial communities, potentially enabling non-pathogenic commensals to become pathogenic. Specifically, in cirrhosis, elevated salivary total protein and immunoglobulin A increase pH levels [81], and oral antibiotics like norfloxacin directly kill Gram-negative bacteria—both disfavoring red complex colonization (attachment and persistence in periodontal pockets) [81,106]. Additionally, cirrhosis-induced innate immune dysfunction impairs local neutrophil function, releasing low-concentration ROS and increasing susceptibility to periodontal pathogens [81,107].

Animal experiments have shown that injecting subgingival plaque from periodontitis patients into the oral cavity of ligatured mice leads to increased CCL4-induced fibrosis in liver tissue sections [75]. The follow-up survey revealed that severe periodontitis was significantly associated with liver cirrhosis-related mortality (hazard ratio (HR) = 2.29) [76]. Both liver cirrhosis and post-liver transplantation patients showed significantly deeper periodontal pocket depth and greater CAL than those of healthy controls. However, cyclosporine A used by liver transplant recipients causes gingival fibroblast hyperproliferation, increased IL-6 secretion, and enhanced collagen synthesis, leading to gingival hyperplasia and compromising periodontal assessment [77,108]. To address this limitation, a study confirmed that periodontal therapy altered salivary microbiota in cirrhotic patients: *Enterobacteriaceae*, *Enterococcaceae*, and *Clostridiaceae* were significantly reduced; these changes were more pronounced in hepatic encephalopathy patients, accompanied by decreased serum endotoxin and systemic inflammation. In contrast, the untreated group showed no salivary changes demonstrated increased endotoxin levels in the blood over the same time period [78].

Collectively, current evidence indicates a significant epidemiological association between periodontitis and the progression of cirrhosis, though further research is warranted to confirm this relationship.

### 2.4. Periodontitis and Hepatocellular Carcinoma

HCC, a primary liver cancer predominantly linked to cirrhosis, includes several established risk factors, including smoking, alcohol, and obesity [109]. Pathologically, it shows ill-defined cell borders and high heterogeneity. Epidemiological trends reveal a >10-fold rise in MASLD-associated HCC, though ALD and hepatitis remain significant risk factors [110].

Recent studies have increasingly revealed that periodontitis is involved in the pathological progression of HCC. Bacteriological detection has shown that patients with MASH-related HCC had significantly higher *P. gingivalis* and *F. nucleatum* titers in gingival crevicular fluid than those with MASH alone [82]. A subsequent cross-sectional study also confirmed this conclusion [111]. *P. gingivalis* may promote rather than initiate HCC development. Supporting this hypothesis, an animal experiment showed that, in MASH mice, *P. gingivalis*-infected liver tumor nodules showed greater tumor size and area, more severe lobular inflammation, and greater hepatocyte balloon degeneration than did the non-infected group [83]. Moreover, a prospective cohort study by Yang has demonstrated that tooth loss correlated positively with HCC development. Losing 11+ permanent teeth or complete permanent tooth loss was linked to HRs of 1.42 and 1.45 for HCC incidence, respectively [84]. However, this study did not exclude the confounding effects of smoking history and age [84]. In addition, Fan utilized bioinformatics, with data derived from the GEO database, to construct a ceRNA (coding or non-coding RNAs that compete for shared miRNAs via common response elements to indirectly regulate other RNA expression) network for periodontitis-related HCC [85]. They identified and screened several genes, such as *mutS* homolog 2 and growth hormone receptor, and verified their prognostic value in HCC [85]. Further experiments are needed to confirm these findings.

In general, current evidence suggests that periodontitis may play a significant role in the progression of HCC, particularly in the context of MASH. The presence of specific periodontal pathogens, such as *P. gingivalis*, appears to exacerbate HCC development and progression. However, further research is needed to elucidate the underlying mechanisms and to address potential confounding factors in epidemiological studies.

The above studies reveal a bidirectional association between periodontitis and chronic liver disease. However, the level of evidence and the reliability of the association vary across different diseases. This article summarizes the existing specific evidence and strength grading (Table 2) to provide a reference for subsequent research.

## 3. The Main Mechanism of Periodontitis Affecting Chronic Liver Diseases

Substantial evidence confirms that periodontitis may contribute to CLDs through multiple mechanisms, including systemic inflammatory responses, impaired hepatic parenchymal and non-parenchymal cell function, and alterations in gut microbiota composition. Additionally, recent studies have further suggested that ferroptosis and immune evasion may also serve as potential pathogenic mechanisms.

### 3.1. Periodontitis Triggers Systemic Inflammation to Drive Chronic Liver Disease Progression

The damaging effect of periodontitis on the liver is mainly facilitated by periodontal pathogens; these pathogens can enter the bloodstream through periodontal pockets or infected pulp chambers and form bacterial colonization in distant organs, including the liver [2,5,112]. The main pathways involved include the direct effects of inflammatory cytokines on hepatic cellular function and synergy between inflammatory cytokines and oxidative stress responses.

In terms of signaling pathways, the core pathways of liver inflammation induced by *P. gingivalis* include the Toll-like receptors (TLRs), NF-κB, and the complement activation system, with synergistic effects among these pathways. LPS derived from this pathogen can activate both TLR2 and Toll-like receptor 4 (TLR4), recruiting the key adaptor myeloid differentiation primary response 88 (MyD88) to initiate downstream signaling [11], upregulate the downstream extracellular signal regulated kinase (ERK) and NF-κB pathways, and promote the release of tumor necrosis factor-α [55]. Meanwhile, the TLRs pathway can also synergize with the NOD (nucleotide-binding oligomerization domain)-like receptor family pyrin domain containing 3 (NLRP3) inflammasome to upregulate the expression of liver inflammatory factors such as MCP-1 and IL-17 [47]. In addition, *P. gingivalis* can further activate NF-κB via the MAPK/ERK pathway.

**Figure 2:**
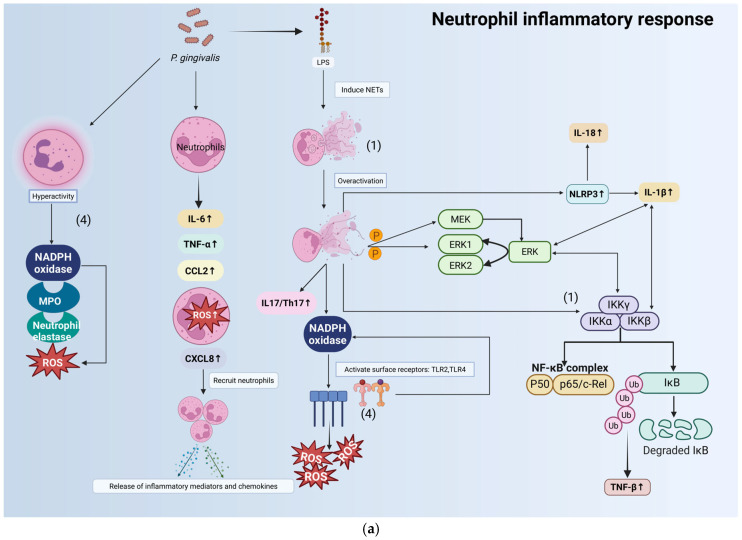

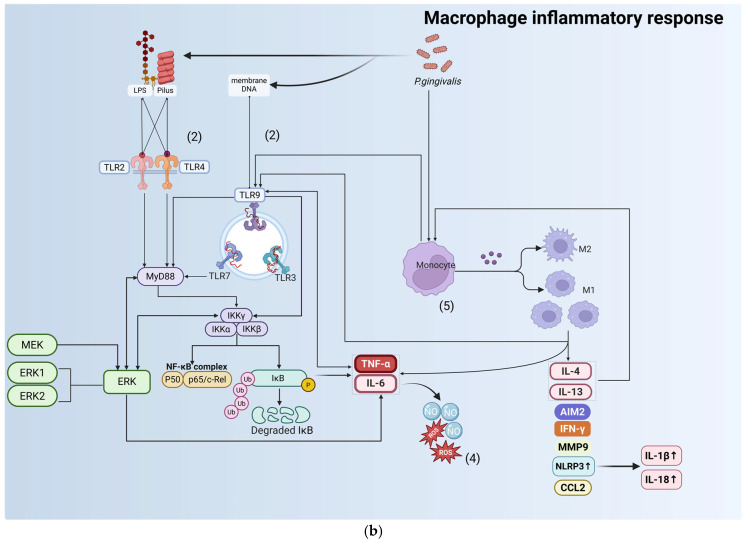

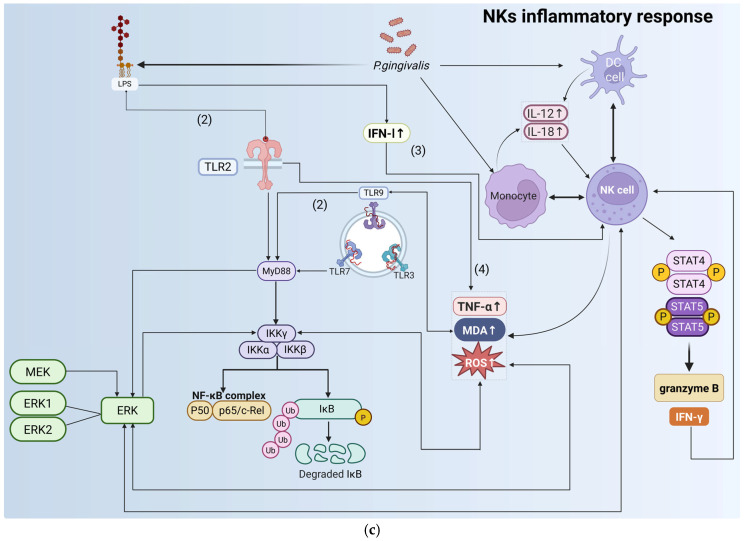
Inflammatory response patterns of neutrophils, macrophages, and natural killer cells in *Porphyromonas gingivalis* infection. (**a**) Neutrophil inflammatory response: *P. gingivalis* activates neutrophils, releasing inflammatory mediators including serine proteases, phosphatase, and ROS; *P. gingivalis*-LPS induces excessive NETs, activating the IL-17/Th17 axis and MEK/ERK phosphorylation to elevate ROS; NETs synergize with NF-κB to upregulate NLRP3/IL-1β, amplifying inflammation. (**b**) Macrophage inflammatory response: *P. gingivalis*-LPS and fimbriae activate TLR2/4, triggering the MEK/ERK and NF-κB signaling pathways; *P. gingivalis*-membrane DNA binds TLR9; excessive NO and intracellular ROS are generated; *P. gingivalis* induces macrophage M1 polarization, driving the release of pro-inflammatory mediators. (**c**) NK cells inflammatory response: *P. gingivalis*-LPS binds TLR2, activating inflammatory signaling pathways or upregulating IFN-I, thereby promoting NKs activation; *P. gingivalis* enhances interactions between macrophages, DCs, and NKs, amplifying inflammatory cascades.All arrows in this figure represent cascade reactions or downstream products. Abbreviations: Th17, T helper 17 cell; ERK1, extracellular signal-regulated kinase 1; ERK2, extracellular signal-regulated kinase 2; MPO, myeloperoxidase; AIM2, absent in melanoma 2; MDA, malondialdehyde; STAT4, signal transducer and activator of transcription 4; STAT5, signal transducer and activator of transcription 5; MEK, mitogen-activated protein kinase kinase; IKKα, inhibitor of κB kinase alpha; IKKβ, inhibitor of κB kinase beta; IKKγ, inhibitor of κB kinase gamma; NLRP3, NLR family pyrin domain containing 3; Ub, ubiquitin; IκB, inhibitor of nuclear factor κB; p50, nuclear factor NF-κB p50 subunit; p65/c-Rel, nuclear factor NF-κB p65 subunit/proto-oncogene c-Rel; NETs, neutrophil extracellular traps; IL-18, interleukin-18; IL-4, interleukin-4; IL-10, interleukin-10; IL-12, interleukin-12; TGF-β, transforming growth factor beta; TNF-β, tumor necrosis factor beta; IFN-I, type I interferon; DC, dendritic cell; NK, natural killer cell; TLR3/4/7/9, Toll-like receptor 3/4/7/9; MyD88, myeloid differentiation primary response 88. Created with BioRender.com and based on the work of [9,115,117,120,121].

The activation of the aforementioned signaling pathways can also regulate the functions of immune cells (neutrophils, macrophages, and natural killer (NK) cells), which in turn can be activated by or provide feedback to these pathways to participate in the progression of liver disease. *P. gingivalis* can induce abnormal activation of these immune cells through direct stimulation or release of virulence factors, leading to massive pro-inflammatory cytokines secretion and systemic inflammatory cascade initiation (Figure 2a–c). Specifically, these processes include neutrophil activation, macrophage M1 polarization, and enhanced interaction among macrophages, dendritic cells (DCs), and NK cells, which secrete inflammatory mediators [113,114,115,116,117].

In addition, the key virulence factors of *P. gingivalis* can induce inflammatory cascade through multiple pathways, as follows: (1) inducing the formation of neutrophil extracellular traps (NETs), synergizing with the NF-κB pathway [118,119]; (2) activating TLR2/TLR4 to trigger mitogen-activated protein kinase kinase(MEK/MKK)/ERK and NF-κB inflammatory signals [9,120,121]; (3) upregulating type I interferon (IFN-I) to promote NK cell activation [120]; (4) increasing the production of intracellular ROS, synergizing with mitochondrial DNA to activate Toll-like receptor 9 (TLR9), thereby further exacerbating oxidative stress [115,119,121]; and (5) promoting M1 macrophage polarization and releasing pro-inflammatory cytokines [117]. These pathways play a critical role in regulating inflammation and cell survival (Figure 2).

Similarly, *F. nucleatum* promotes hepatic inflammatory responses through multiple pathways: it downregulates silent information regulator 1 and adenosine monophosphate-activated protein kinase signaling by inhibiting nicotinamide adenine dinucleotide synthesis, leading to ROS release [13]; additionally, it activates the phosphatidylinositol 3-kinase (PI3K)/protein kinase B (Akt)/mammalian target of the rapamycin pathway [122], further exacerbating the inflammatory microenvironment [13,122].

Abundant evidence supports the idea that periodontitis compromises liver health via systemic inflammation, with modulation of inflammatory cytokines emerging as a viable therapeutic approach to mitigate periodontitis-induced systemic complications.

### 3.2. Periodontitis Impairs Hepatic Cellular Function to Worsen Chronic Liver Dysfunction

The liver comprises diverse cell types, each playing unique and critical roles. Major cell populations include hepatocytes, hepatic stellate cells (HSCs), liver sinusoidal endothelial cells (LSECs), Kupffer cells (KCs), and biliary epithelial cells.

Hepatocytes, the liver’s main functional cells, account for over 70% of liver volume and regulate metabolic, synthetic, and immune processes. Their activity is essential for these functions. *P. gingivalis* and its key virulence factors suppress hepatocyte activity and exacerbate acute and chronic liver injury through multiple mechanisms [44,123,124,125,126,127]. On one hand, its lipopolysaccharide component binds to specific domains on the hepatocyte membrane, enhancing LPS–hepatocyte interactions and inducing abnormal lipid droplet proliferation [124]; on the other hand, it triggers endoplasmic reticulum stress and mitochondrial damage [123,125], causing organelle dysfunction characterized by decreased mitochondrial density [123], disorganized cristae [123], and abnormal glycogen synthesis in the smooth endoplasmic reticulum [123], while activating the NF-κB pathway, leading to hepatocyte inactivation [126]. Notably, the mitochondrial DNA (mtDNA) damage mechanism induced by ROS overproduction in MASH shares similarities with the pathway through which *P. gingivalis* utilizes ROS to propagate inflammation, suggesting mitochondrial dysfunction as a common mechanism. Although molecular markers of endoplasmic reticulum (ER) stress and mitochondrial DNA dysregulation have been well documented [126,127], the ultrastructural alterations in human hepatocytes mediated by *P. gingivalis* and their specific molecular mechanisms remain largely unexplored.

Reduced hepatocyte activity, associated with metabolic dysregulation and oxidative stress, leads to various forms of cellular degenerations including steatosis, hydropic change, hyaline degeneration, and necrosis. *P. gingivalis* primarily exacerbates these changes rather than initiating them. In the context of pre-existing liver disease, *P. gingivalis* infection aggravates hepatocyte steatosis and lipid deposition, induces hydropic changes such as ballooning degeneration, and further activates inflammatory responses, tissue damage, and fibrotic processes [43,55,58]. Additionally, some studies suggest direct hepatotoxic effects of *P. gingivalis* and *F. nucleatum*, which can induce mild steatosis manifested as microvesicular small lipid droplets [128]. Notably, interventions targeting *P. gingivalis* (such as improved oral hygiene or antibiotic treatment) significantly improve these injuries [59,128,129]. Overall, these findings confirm that *P. gingivalis* reduces hepatocyte activity and exacerbates liver pathology.

**Figure 3:**
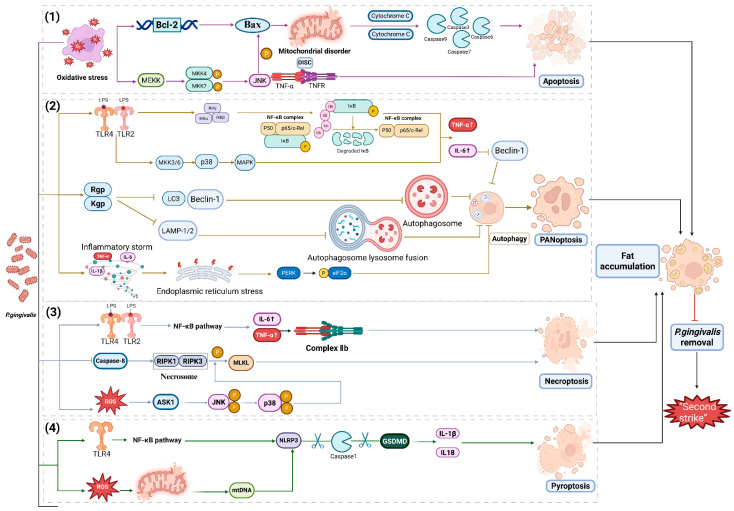
*Porphyromonas gingivalis*-mediated multimodal hepatocyte death: apoptosis, PANoptosis, necroptosis, and pyroptosis. Distinct death models are color-coded, as follows: purple lines (apoptosis), yellow lines (autophagy inhibition and PANoptosis), green lines (necroptosis), and blue lines (pyroptosis). In this figure, arrows indicate cascade reactions or downstream products, and lines with termination bars represent inhibitory effects.(1) Apoptosis (in order to maintain the stability of the internal environment, cells controlled by genes die spontaneously and in an orderly manner). *P. gingivalis* induces oxidative stress, triggering Bcl-2-mediated Bax activation, mitochondrial membrane damage, cytochrome c release, and caspase activation to orchestrate hepatocyte apoptosis; *P. gingivalis* activates the MKK-JNK cascade to promote Bax phosphorylation, while TNF-α and TNFR interaction forms the DISC, promoting hepatocyte apoptosis. (2) Autophagy (a process in which cells engulf cytoplasmic proteins or organelles, package them into vesicles, and fuse with lysosomes to form autolysosomes for degradation of the sequestered contents inhibition) and PANoptosis (an inflammatory form of programmed cell death regulated by the PANoptosome complex, featuring key characteristics of pyroptosis, apoptosis, and/or necroptosis). *P. gingivalis*-LPS binds TLR2/4, activating the NF-κB/MAPK pathways to upregulate pro-inflammatory cytokines while suppressing autophagy initiation via LC3 downregulation; *P. gingivalis*-derived proteases degrade LAMP1/2, preventing autophagosome–lysosome fusion and terminating autophagic flux; *P. gingivalis*-induced inflammatory cascades trigger ER stress, activating the PERK/eIF2α pathway to transcriptionally repress autophagy-related genes. (3) Necroptosis (when apoptosis is blocked, necroptosis is the process of self-destruction of cells activated by extracellular signals or intracellular signals). *P. gingivalis*-LPS specifically binds to TLR2/TLR4, activates NF-κB, releases inflammatory mediators, upregulates Complex IIB, and promotes necroptosis; *P. gingivalis* inhibits caspase-8, activates the necrosome, upregulates ROS, regulates the ASK1/JNK pathway, phosphorylates MLKL, and promotes necroptosis. (4) Pyroptosis (programmed cell death mediated by gasdermin protein and characterized by cell membrane perforation, content release, and inflammatory response activation). *P. gingivalis* activates the TLR4/NF-κB pathway and inducing ROS production, which triggers mitochondrial dysfunction and mtDNA release. The leaked mtDNA activates the NLRP3 inflammasome, leading to caspase-1 cleavage. Active caspase-1 then incises GSDMD, releasing pro-inflammatory mediators and executing pyroptosis. Abbreviations: TNFR, tumor necrosis factor receptor; MEKK, mitogen-activated protein kinase kinase kinase; MKK4, mitogen-activated protein kinase kinase 4; MKK7, mitogen-activated protein kinase kinase 7; MKK3, mitogen-activated protein kinase kinase 3; MKK6, mitogen-activated protein kinase kinase 6; Rgp, Arg-gingipain; RIPK3, receptor-interacting protein kinase 3; RIPK1, receptor-Interacting protein kinase 1; Bcl-2, B-cell lymphoma 2; Bax, BCL2-associated X protein; JNK, c-Jun N-terminal kinase; DISC, death-inducing signaling complex; p38, p38 mitogen-activated protein kinase; MAPK, mitogen-activated protein kinase; LC3, microtubule-associated protein 1 light chain 3; LAMP-1/2, lysosomal-associated membrane protein 1/2; PERK, protein kinase R-like ER kinase; eIF2α, eukaryotic translation initiation factor 2α; MLKL, mixed lineage kinase domain-like protein; ASK1, apoptosis signal-regulating kinase 1; mtDNA, mitochondrial DNA; GSDMD, gasdermin D. Created with BioRender.com and based on the work of [125,127,130,132,133,134,135,136,137,138].

Mild hepatocyte degeneration is usually reversible with functional restoration, whereas severe degeneration leads to cell death. In addition to ferroptosis, mentioned earlier, hepatocytes may undergo necroptosis, apoptosis, pyroptosis, and PANoptosis [83,125,127,130,131,132,133,134,135]. Notably, emerging evidence identifies *P. gingivalis* as a key orchestrator of these hepatocyte death modalities. It and its virulence factors (e.g., LPS, gingipains) trigger a cascade of cell death pathways through various mechanisms (Figure 3). (1) By activating the B-cell lymphoma 2 (Bcl-2)/Bcl-2-associated X protein (Bax) pathway, the MKK-c-Jun N-terminal kinase (JNK) pathway, releasing cytochrome c and forming the death-inducing signaling complex (DISC), *P. gingivalis* induces apoptosis [130,134]. (2) By activating the NF-κB/MAPK and protein kinase R-like endoplasmic reticulum kinase (PERK)/eukaryotic initiation factor 2α (eIF2α) pathways and downregulating microtubule-associated protein 1 light chain 3 (LC3) and lysosomal-associated membrane protein 1/2 (LAMP1/2), *P. gingivalis* inhibits autophagy and promotes PANoptosis [127,132,133,136,137,138]. (3) By activating the NF-κB and apoptosis signal-regulating kinase 1 (ASK1)/JNK pathways, releasing complex IIB and phosphorylating mixed lineage kinase domain-like protein (MLKL), *P. gingivalis* promotes necroptosis [125]. (4) By activating the NF-κB pathway and eliciting mitochondrial dysfunction, cleaving gasdermin D (GSDMD) and releasing pro-inflammatory mediators, *P. gingivalis* facilitates pyroptosis [135].

**Figure 4:**
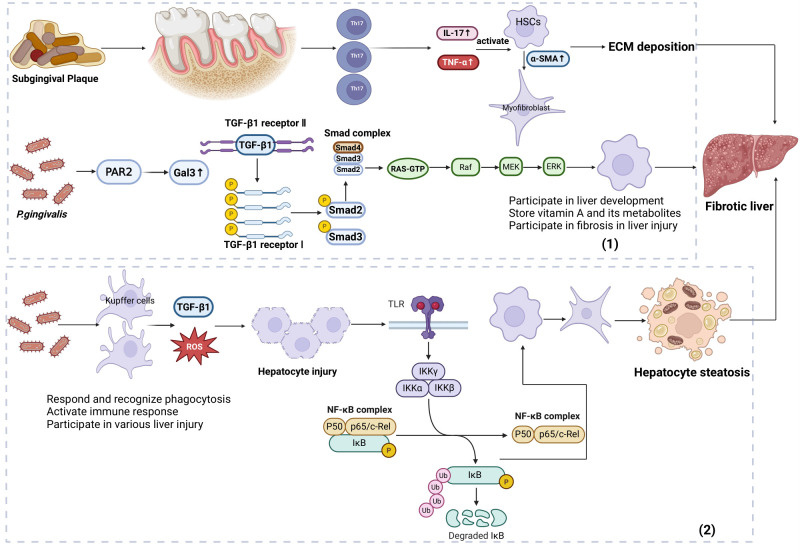
*Porphyromonas gingivalis*-mediated multimodal hepatocyte death: apoptosis, PANoptosis, necroptosis, and pyroptosis. (1) HSCs: Participate in liver development, store vitamin A and metabolites, activate and drive fibrosis during liver injury; subgingival plaque induces Th17 expansion, secreting IL-17/TNF-α to promote HSCs differentiation into myofibroblasts (alpha smooth muscle actin (α-SMA) ↑, ECM ↑) and fibrosis; *P. gingivalis* directly stimulates HSCs via Gal-3-mediated TGF-β1/Smad2/ERK signaling, exacerbating hepatic fibrosis. (2) KCs: Initiate immune responses (pathogen recognition, phagocytosis, inflammation) during infection and contribute to acute/chronic liver injury; *P. gingivalis* infection increases KCs numbers, secreting TGF-β1 and ROS to damage hepatocytes and activate HSCs, promoting steatosis and fibrosis; no direct clinical evidence links KCs to human hepatic fibrosis. All arrows in this figure represent cascade reactions or downstream productsAbbreviation: Smad3, SMAD family member 3; Smad4, SMAD family member 4; Smad2, SMAD family member 2; PAR2, protease-activated receptor 2; Gal3, galectin-3; RAS-GTP, RAS-guanosine triphosphate; Raf, RAF proto-oncogene serine/threonine-protein kinase; HSCs, hepatic stellate cells; α-SMA, alpha-smooth muscle actin; TGF-β1 receptor I, transforming growth factor beta 1 receptor type I; TGF-β1 receptor II, transforming growth factor beta 1 receptor type II. Created with BioRender.com and based on the work of [44,49,75,139,140,141,142,143,144,145,146].

HSCs and KCs play a pivotal role in the periodontal pathogen-induced fibrotic cascade (Figure 4). This process involves the following: (1) Subgingival plaque induces Th17 cell expansion, and the secreted IL-17/TNF-α activates the NF-κB/MAPK signaling pathways in HSCs to regulate transcription factors such as signal transducer and activator of transcription 3 (STAT3), thereby promoting HSCs differentiation into myofibroblasts [75,139]. Additionally, *P. gingivalis* mediates protease-activated receptor 2 (PAR2) activation on the HSCs, which triggers the autocrine release of transforming growth factor-beta 1 (TGF-β1) and the upregulation of galectin-3 (Gal-3) in HSCs, subsequently activating the TGF-β1/SMAD family member 2 (Smad2)/ERK signaling pathway to induce the myofibroblastic activation of HSCs and further exacerbate hepatic fibrosis [140,141,142]. (2) *P. gingivalis* increases the number of KCs, which in turn release TGF-β1 and ROS; ROS initiates oxidative stress to damage hepatocytes and together with TGF-β1, promotes hepatic steatosis and fibrosis [44,49,143,144,145], although there is no direct clinical evidence linking KCs to liver fibrosis in humans [146].

Current research has established compelling evidence that periodontitis impairs hepatic parenchymal and non-parenchymal cell function through multiple interconnected pathways, thereby exacerbating the progression of CLDs. These findings collectively suggest that restoring hepatic cellular homeostasis offers therapeutic potential for mitigating periodontitis-associated liver injury.

### 3.3. Periodontitis Modulates the Oral–Gut–Liver Axis to Accelerate Hepatic Pathology

The impact of *P. gingivalis* on systemic diseases through modulation of the gut microbiota has received significant attention, particularly within the oral–gut–liver axis framework. The gut-liver axis, first proposed by Marshall, emphasizes the bidirectional interactions between the gut and the liver in disease pathogenesis [147]. Subsequent studies have extended this concept to the oral–gut–liver axis, linking oral microbes to CLDs. It has been reported that oral pathogens disrupt the gut microbiota, impair intestinal barrier function and immune regulation, and enable bacterial toxins and inflammatory cells to translocate to the liver via the portal vein [148,149]. This pathway manifests functionally as significant alterations in fecal metabolites, accompanied by increased hepatic lipid deposition [150,151], while these pathogens exacerbates chronic liver disease through gut dysbiosis and barrier disruption [67].

Studies have revealed that *P. gingivalis* infection significantly alters gut microbiota composition, as primarily characterized by an increased *Firmicutes*/*Bacteroidetes* ratio, elevated *Firmicutes* abundance, enriched *Prevotella* proportions, and a significant reduction in beneficial bacteria (such as *Bifidobacterium* and *Lactobacillus*) [152]. Although some studies reported opposing findings [151], the overall evidence supports that *P. gingivalis* infection induces an elevated *Firmicutes*/*Bacteroidetes* ratio and the depletion of beneficial bacteria [152]. Multi-species infection (*P. gingivalis*, *F. nucleatum*, and *T. forsythia*) further exacerbates microbiota dysbiosis [9], specifically resulting in elevated *T. forsythia* levels in the feces and liver [111] and significant enrichment of *P. gingivalis* and *F. nucleatum* in the gut [9], with concurrent suppression of *Bifidobacterium* and *Lactobacillus*. Similar patterns are also observed in other experiments [153].

These microbiota alterations directly impair the gut immune protective effect on the liver. Butyrate produced by *Firmicutes* normally alleviates hepatic inflammation, but dysbiosis leads to altered fatty acid metabolism, which disrupts hepatic lipid metabolism and promotes increased hepatic lipid deposition [154].

Furthermore, *P. gingivalis* and its virulence factors (particularly LPS and proteases) downregulate tight junction proteins (such as zonula occludens-1 and occludin (OCLN)) [155], impair intestinal epithelial barrier integrity, and markedly increase permeability. This results in colonic structural damage, increased inflammatory cell infiltration, and substantial reduction of the mucus layer [156], thereby enabling these pathogens and their virulence factors to translocate to the liver via the portal vein. *F. nucleatum* alone, as well as biofilm experiments, consistently demonstrate the synergistic occurrence of intestinal barrier disruption and microbiota dysbiosis, collectively accelerating the pathological progression of chronic liver disease [152,157].

**Figure 5:**
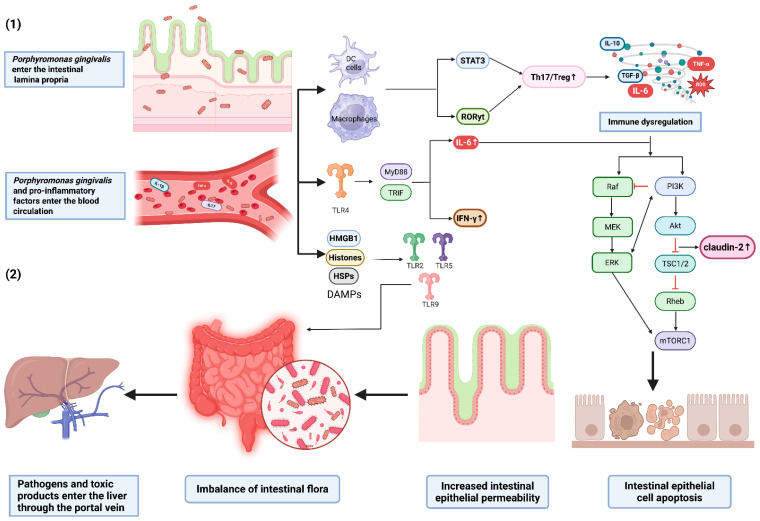
*Porphyromonas gingivalis* translocation via the oral–gut–liver axis: mechanisms of gut dysbiosis and inflammatory synergy. (1) *P. gingivalis* activates TLR4 signaling, promoting IL-6/IFN-γ secretion and exacerbating Th17/Treg imbalance via STAT3/RORγt signaling, thereby aggravating intestinal inflammation, and releases DAMPs (HMGB1, histones, HSPs) to activate TLR2, TLR5, and TLR9 expression, exacerbating local inflammation. (2) IL-6 upregulates claudin-2 (via MEK/ERK and PI3K pathways), increasing intestinal permeability, while TNF-α/ROS-induced epithelial apoptosis synergistically disrupts gut microbiota homeostasis; pathogens and toxic products enter the liver via the portal vein. In this figure, arrows indicate cascade reactions or downstream products, and lines with termination bars represent inhibitory effects. Abbreviations: Treg, regulatory T cell; TRIF, TIR-domain-containing adapter-inducing interferon-β; TSC1/2, tuberous sclerosis complex 1/2; Rheb, Ras homolog enriched in brain; mTORC1, mechanistic target of rapamycin complex 1; HMGB1, high-mobility group box 1 protein; HSPs, heat shock proteins; STAT3, signal transducer and activator of transcription 3; RORγt, retinoic acid-related orphan receptor gamma t; PI3K, phosphatidylinositol 3-kinase; Akt, AKT serine/threonine kinase; DAMPs, damage-associated molecular patterns; TLR5, Toll-like receptor 5. Created with BioRender.com and based on the work of [158,159,160,161,162].

The molecular mechanisms by which *P. gingivalis* translocate to the liver via the oral–gut–liver axis have been further elucidated. *P. gingivalis* activates TLR4 and its downstream STAT3/retinoic acid receptor-related orphan receptor gamma t (RORγt) signaling pathway [158], inducing IL-6 release and thereby increasing gut permeability. Concurrently, *P. gingivalis*-induced ROS promote intestinal epithelial cell apoptosis [159], further disrupting the intestinal physical barrier and enabling microbial invasion of the mucosal layer, which fosters an inflammatory microenvironment. On one hand, this process releases damage-associated molecular patterns (DAMPs) [160], activating TLR2, Toll-like receptor 5 (TLR5), and TLR9 expression in the intestinal mucosa and exacerbating local inflammation [161]; on the other hand, it drives the gut microbiota shift from a Gram-positive-dominated beneficial community to a Gram-negative-dominated population, promoting the proliferation of facultative anaerobic pathogens [162]. These mechanisms interact synergistically with the aforementioned pathways of gut dysbiosis and barrier disruption, collectively facilitating the systemic translocation of periodontal pathogens to the liver (Figure 5).

Therefore, interventions targeting gut dysbiosis and intestinal barrier impairment have also emerged as potential therapeutic targets for adjuvant therapy for CLDs [106]. Specifically, probiotics (e.g., *Lactobacillus* and *Bifidobacterium*) can effectively improve liver injury by restoring intestinal barrier integrity via upregulation of OCLN, suppressing miR-122a and reducing Gram-negative aerobic bacterial loads [163,164,165]. Therefore, supplementation with specific probiotics in patients with periodontitis may represent a therapeutic target to slow the progression of CLDs.

### 3.4. Periodontitis Induces Ferroptosis to Exacerbate Chronic Liver Injury

Ferroptosis is a non-apoptotic cell death characterized by lipid peroxidation and iron accumulation, leading to plasma membrane damage and regulated necrosis. This process is fundamentally driven by an imbalance in redox homeostasis [166]. Current studies classify its regulatory factors into three categories: (1) iron metabolism-related factors, e.g., transferrin, transferrin receptor, etc. [167]; (2) lipid peroxidation regulators, e.g., lipoxygenases, nicotinamide adenine dinucleotide phosphate (NADPH) oxidase, etc. [168]; and (3) antioxidant systems, e.g., glutathione peroxidase 4 (GPX4), ferroptosis suppressor protein 1/coenzyme Q10, etc. [169].

The damage caused by ferroptosis is not confined to local areas. On one hand, it elevates systemic oxidative stress [170]; on the other hand, it disrupts iron homeostasis, increases hepcidin expression [171], and impairs the uptake and degradation of transferrin, thereby reducing circulating iron levels while promoting intracellular iron overload [172], ultimately exacerbating multi-organ dysfunction.

Emerging evidence indicates a significant link between periodontitis, ferroptosis, and liver disease, suggesting that periodontitis may promote the progression of chronic liver disease by inducing and exacerbating cellular ferroptosis [44,45,46,173,174].

Current research indicates that *P. gingivalis* induces hepatocyte ferroptosis and accelerates liver injury progression through three independent pathways. *P. gingivalis* activates the NF-κB signaling pathway in the hepatocytes, transcriptionally upregulating pro-ferroptotic molecules such as acyl-CoA synthetase long-chain family member 4 (ACSL4) and heme oxygenase-1, while downregulating key ferroptosis inhibitors solute carrier family 7 member 11 (SLC7A11) and GPX4, thereby promoting lipid peroxidation accumulation and iron-dependent cell death [44,46]. Other studies show that *P. gingivalis* infection suppresses the glutamate synthesis pathway, triggering ferroptosis, and the ferroptosis-specific inhibitor ferrostatin-1 significantly reverses *P. gingivalis*-induced hepatocyte injury, inflammation, and steatosis, confirming ferroptosis as a critical pathway in *P. gingivalis*-promoted liver damage [45,173]. In the IL-6/STAT3/hepcidin axis-driven iron overload pathway, *P. gingivalis* activates this signaling cascade to upregulate the transferrin receptor and downregulate the iron exporter solute carrier family 40 member 1, leading to increased hepatic iron accumulation, and the inhibition of STAT3 markedly alleviates the iron metabolism disorder and downstream ferroptosis [174].

These findings suggest that periodontitis may promote liver injury by inducing ferroptosis (particularly in hepatocytes), but the exact relationship and underlying molecular mechanisms require further in-depth study. Monitoring ferroptosis-related biomarkers (such as ACSL4 and SLC7A11) in periodontitis patients, combined with inhibitor interventions to block this process [175,176], may help reduce the systemic adverse effects of oral inflammation and offer new therapeutic strategies.

### 3.5. Periodontitis Mediates Pathogen Immune Evasion to Promote Chronic Liver Damage

Immune evasion refers to the ability of pathogens and other entities to evade recognition and clearance by the immune system through various mechanisms. Recent studies have shown that *P. gingivalis* can evade adaptive immune responses by impairing the functions of multiple immune cells [177,178].

Notably, the periodontal pathogen *P. gingivalis* disrupts immune responses by targeting immune cells and key inflammatory mediators to enable immune evasion. Its virulence factors employ highly specific strategies.

Minor fimbriae and OMVs target DC-specific intercellular adhesion molecule-3-grabbing non-integrin to evade DC autophagy and cytotoxic killing, interfere with antigen processing and presentation, downregulate major histocompatibility complex class II (MHC-II) expression, block Bcl-2-interacting protein 1/PI3K/autophagy-related protein 9 and MAPK/ERK pathways, and transmit immunosuppressive signals to T cells, NK cells, and other immune cells [179,180]. FimA fimbriae activate TLR2 and C-X-C chemokine receptor type 4 (CXCR4) to suppress interleukin production, impair phagocytosis by liver macrophages, downregulate cluster of differentiation 86 and cluster of differentiation 40, inhibit cluster of differentiation 4 positive (CD4^+^) and cluster of differentiation 8 positive (CD8^+^) T cell activation, and block recognition and clearance of *P. gingivalis* [181,182].

The capsule enables immune evasion through a multi-layered strategy. First, it utilizes negative electrostatic repulsion to prevent NETs from adsorbing *P. gingivalis*, while surface modifications simultaneously weaken NET recognition and capture efficiency [19]. Upon escaping NET entrapment, *P. gingivalis* further suppresses neutrophil function by inhibiting NADPH oxidase activation [183]. Additionally, proteases orchestrate immune evasion through diverse mechanisms: They upregulate interleukin-10 (IL-10) expression and suppress antigen-presenting cell function to hinder T cell activation [184]; inhibit the PI3K-Akt pathway in B cells, thereby reducing IL-6 production, downregulating B10 cells (a major Breg subset) and upregulating CD19-positive regulatory B cell (CD19^+^ Breg) levels to promote Treg activation [185,186]; activate TLR2-PI3K pathways in macrophages and neutrophils to block lysosomal fusion and evade phagocytosis [187,188]; and directly kill or degrade cytokines [149].

Furthermore, *P. gingivalis* modulates immune function through additional pathways: Its supernatant significantly downregulates CD4^+^ and CD8^+^ T cell expression, suppressing T cell activation [162]; in stage II periodontitis patients, it promotes CD19^+^ Breg cell expansion in peripheral blood and inflamed gingival tissue, which is positively correlated with IL-10 and transforming growth factor-beta (TGF-β) levels [189,190]; it activates the TGF-β pathway to drive Treg hyperactivation, impairing effector T cell and NK cell function and reducing pathogen recognition and clearance; it forms biofilms that synergize with capsule and LPS to inhibit NET recognition [183]; and it binds erythrocytes while aberrantly activating complement receptor 3, upregulating IL-10 and TGF-β to induce immunosuppressive function in liver Kupffer cells and macrophages [191]. The key strategies include inhibiting immune cell activation, impairing antigen presentation, and modulating signaling pathways (Table 3).

The liver, characterized by its immune-tolerant properties, is a common site where pathogens evade immune surveillance [192]. HBV and HCV evade immune recognition by suppressing MAPK/ERK phosphorylation in KCs, reducing NET release, and enhancing Treg activity, thereby promoting viral replication and increasing the risk of fibrosis and HCC [193,194]. Furthermore, gut-derived bacterial LPS exacerbates immune evasion by impairing CD4^+^ and CD8^+^ T cell effector functions, contributing to liver fibrosis [195]. The PD-1/PD-L1 axis, a critical regulator of hepatic immunity, is markedly upregulated in HBV infection [196], and the targeted blockade thereof demonstrates therapeutic promise for HBV infection, HCC, and NAFLD [196,197,198,199]. Notably, these molecular pathways constituting the hepatic immune evasion microenvironment (programmed cell death protein 1 (PD-1)/programmed death-ligand 1 (PD-L1), complement system, and NET dysfunction) can likewise be exploited by *P. gingivalis* [116,200,201]. However, direct evidence for *P. gingivalis* initiating hepatic immune evasion through these pathways remains limited, requiring further investigation.

*P. gingivalis* promotes the progression of chronic liver disease through multiple immune escape mechanisms. It utilizes OMVs carrying gingipains to evade immune recognition, to disseminate to the liver, and to accumulate there, thereby directly impairing hepatic metabolic function [48]. Its LPS and virulence factors suppress macrophage antigen presentation by downregulating related genes such as C-type lectin domain family 20 member A, histocompatibility 2, D Region, member B1; and histocompatibility 2, A Region, member A, reducing pathogen clearance efficiency and prolonging the duration of pathogen action [49]. Regarding adaptive immunity, *P. gingivalis* disrupts Th17/Treg homeostasis, promotes CD4^+^ T cell differentiation toward Th17 (with upregulation of IFN-γ and the signal transducer and activator of transcription 1), and reduces Treg cell numbers, thereby impairing immunosuppressive function and exacerbating hepatic inflammation [47,51]. Furthermore, this immune escape exhibits genetic susceptibility: in the context of nuclear factor erythroid 2-related factor 2 gene deficiency, *P. gingivalis* LPS upregulates lipopolysaccharide-binding protein and aggravates fibrosis [50], suggesting a synergistic interaction between immune escape and systemic oxidative stress.

Studies have confirmed that other key periodontal pathogens can also aggravate liver injury through immune escape mechanisms: the major sheath protein of *T. denticola* interferes with neutrophil signaling pathways, activates phosphatase and tensin homolog, inhibits PI3K/Akt and neutrophil polarization, and weakens chemotaxis [202]. The S-layer protein of *T. forsythia* weakens the immune response of liver macrophages and inhibits the expression of IL-1β and TNF-α [202]. Meanwhile, its metalloproteinase mirolysin blocks the deposition of complement C3b/C4b, reducing pathogen clearance [203]. Similarly, *F. nucleatum* promotes M2 polarization of macrophages, inhibits the activation of cluster of differentiation 3 positive, CD4^+^, and CD8^+^ T cells, and thereby suppresses hepatic adaptive immunity [12,204].

The compelling evidence outlined above indicates that immune evasion represents a central mechanism whereby periodontitis exacerbates chronic hepatic injury, involving innate immunity, adaptive immunity, and other pathways. Targeting the PD-1/PD-L1 axis with inhibitors [205], modulating the Th17/Treg balance [206], or targeting NET modulators may provide novel therapeutic avenues for preventing periodontal disease-associated liver disease progression [207].

### 3.6. Periodontitis Potentiates Endothelial-to-Mesenchymal Transition to Exacerbate Chronic Hepatic Inflammation

**Figure 6:**
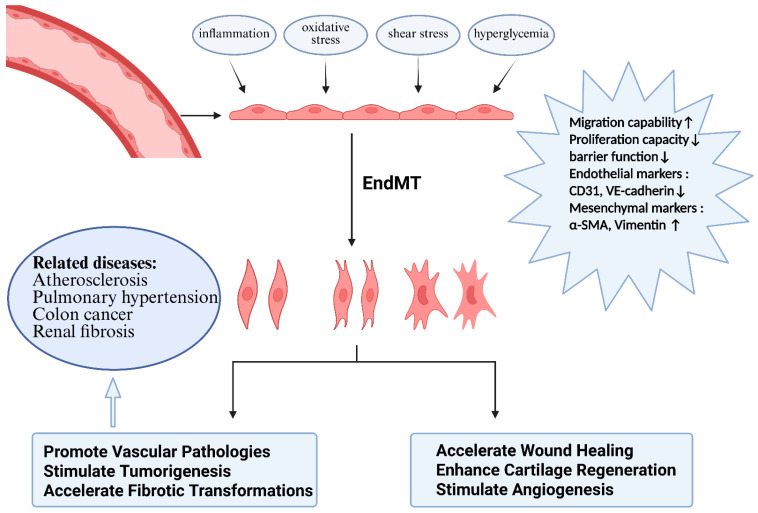
Endothelial–mesenchymal transition: triggers, phenotypic switching and fulfills dual roles. EndMT is characterized by downregulation of endothelial markers (CD31) and vascular endothelial cadherin (VE-cadherin), upregulation of mesenchymal markers (α-SMA, vimentin), and functional alterations in cells. Triggered by inflammatory cytokines, oxidative stress, shear stress, or hyper-glycemia, EndMT exhibits dual roles, i.e., promoting tissue repair (wound healing, angiogenesis) while driving pathologies such as fibrosis, atherosclerosis, pulmonary hypertension, and cancer. All arrows in this figure represent cascade reactions. Abbreviation: PECAM-1/CD31, platelet endothelial cell adhesion molecule-1; EndMT, endothelial–mesenchymal transition. Created with BioRender.com and based on the work of [208,209,210,211].

**Figure 7:**
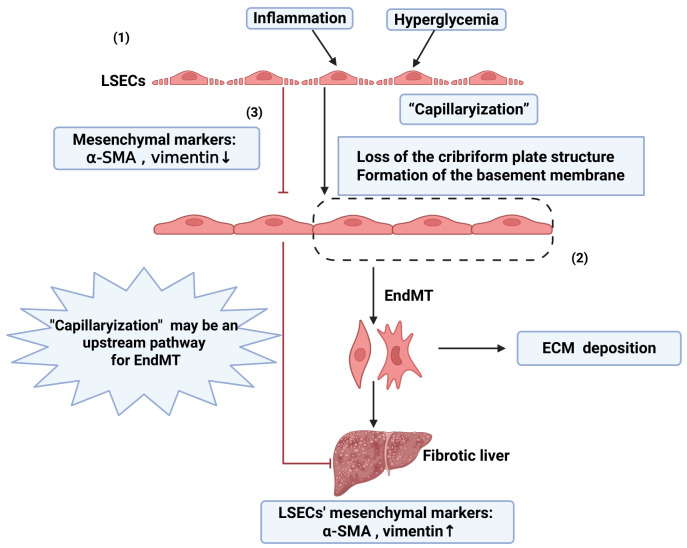
Liver sinusoidal endothelial cell capillarization as a trigger of endothelial–mesenchymal transition in liver fibrosis. (1) Inflammation and hyperglycemia promote LSECs capillarization; (2) isolation of capillarized LSECs demonstrates that a subset undergoes EndMT, resulting in perisinusoidal ECM deposition and aggravated liver fibrosis; (3) blockade of capillarization downregulates mesenchymal markers with concomitant attenuation of hepatic fibrosis. Symbols used in the figure are defined as follows: solid arrows indicate cascade reactions; red blunt-ended lines represent inhibition. Abbreviations: LSECs, liver sinusoidal endothelial cells. Created with BioRender.com and based on the work of [212,213].

Endothelial–mesenchymal transition (EndMT) is a process in which endothelial cells differentiate into mesenchymal cells when stimulated by inflammatory factors, oxidative stress, or shear stress, and this process is accompanied by alterations in cellular markers and functional changes. It exerts dual roles in tissue repair and organ fibrosis and serves as a core mechanism driving the progression of myocardial fibrosis, atherosclerosis, and chronic liver diseases [208,209,210,211] (Figure 6). In the liver, LSECs—a specialized endothelial cell population—undergo capillarization (a loss of fenestrated sieve-like structures and the formation of a basement membrane), which has been identified as a precursor event of EndMT [212,213]. Blocking this process (capillarization or EndMT) inhibits the progression of hepatic fibrosis (Figure 7).

Current evidence indicates that periodontal and enteric pathogens can promote endothelial dysfunction through the EndMT pathway: *P. gingivalis* has been demonstrated to act as a key inducer activating EndMT in vascular endothelial cells [52], while *Escherichia coli* has also been shown to directly induce EndMT in LSECs and facilitate NAFLD progression [53].

However, although these findings suggest pathological interactions between bacteria and endothelial cells, there is currently no direct evidence demonstrating that *P. gingivalis* can directly induce EndMT in LSECs or exacerbate the progression of human hepatic fibrosis. This research gap remains to be addressed through further in vitro experiments and animal models, which may serve as an entry point for investigating periodontal pathogen-associated hepatic fibrosis.

Based on the mechanistic insights discussed above, this review synthesizes the diverse pathways through which periodontitis contributes to CLDs, integrating relevant experimental and clinical evidence. The identified mechanisms are systematically categorized and presented according to established evidence grading criteria (Table 4).

## 4. Conclusions

Although direct evidence for a causal link between *P. gingivalis* and chronic liver diseases remains limited, numerous studies have confirmed a significant association between periodontitis and CLDs.

Existing evidence on the effects of *P. gingivalis* on CLDs—mediated through systemic inflammation, gut homeostasis disruption, and cellular damage—is relatively robust, but critical and direct evidence for its involvement in ferroptosis and immune evasion remains limited, and that which exists has been derived from animal studies. Moreover, direct evidence in the field of the endothelial–mesenchymal transition is still lacking.

Notably, recent studies have shown that other key periodontal pathogens (such as *T. forsythia*, *F. nucleatum*, and *T. denticola*) can also aggravate liver injury through multiple mechanisms, including immune escape, the oral–gut–liver axis, and systemic inflammatory response, suggesting that the impact of periodontitis on the liver is synergistic with multiple pathogens, rather than the result of *P. gingivalis* acting alone.

Future research should prioritize these areas to gain a more comprehensive understanding of *P. gingivalis* (and other key periodontal pathogens) in regards to hepatic health. Interventions targeting the blockade of *P. gingivalis*-mediated pathways involving immune evasion, ferroptosis, and EndMT may help inhibit the progression of liver diseases. Importantly, periodontitis is not merely a localized oral inflammatory condition but is closely linked to systemic diseases, underscoring the necessity of maintaining good oral health. This connection also provides a novel perspective for the prevention and management of liver diseases.

## Figures and Tables

**Table 2:** Strength of evidence for the association between periodontitis and chronic liver diseases.

Liver Diseases	Highest Evidence Available	Other Supporting Data	Grade	Key Limitations
**MASLD**	Systemic Review and Meta-Analysis [60]	Retrospective Cohort Study [57] Translational Animal Study with Clinical Correlation [56] Animal Experiment [55,58,59,61] Narrative Review [7,62]	Moderate	Meta-analysis based on heterogeneous observational studies without confirmed temporal sequence; no RCTs or prospective cohorts with hard MASLD endpoints.
**Chronic hepatitis**	Cross-Sectional Study [70]	Cross-Sectional Study [64,65,66,67,69] Retrospective Cohort Study [68] Animal experiment [47] Narrative Review [7]	Moderate	Prospective studies are limited to the elderly population and insufficiently account for confounding factors such as age and socioeconomic status.
**Cirrhosis**	Longitudinal Cohort Study [76]	Case-Control Study [72,77] Cross-Sectional Study [74,77,80] Retrospective Cohort Study [63] Observational Clinical Study [78,107] Animal Experiment [75] In Vitro Study [108] Narrative Review [81,106]	Moderate	A single prospective cohort study with mortality as the endpoint, but not targeting liver-specific causes; recall bias and selection bias were not excluded.
**HCC**	Longitudinal Cohort Study [84]	Cross-Sectional Study [82,111] Bioinformatics Analysis Study [85] Animal Experiment [83]	Moderate to Low	Using tooth loss as a proxy indicator for periodontitis without conducting clinical periodontal assessments; cross-sectional design is not conducive to causal inference and determination of temporal sequence.

Abbreviations: MASLD, metabolic dysfunction-associated steatotic liver disease; HCC, hepatocellular carcinoma; RCT, randomized controlled trial.

**Table 3:** Immune evasion mechanisms mediated by key virulence factors of *Porphyromonas gingivalis*.

Virulence Factor/Effector	Targeted Immune Component	Key Findings/Mechanisms	References
**Gingipains**			
**Gingipains**	IL-10	Upregulates IL-10 expression, suppresses antigen-presenting cell function.	[184]
**Gingipains**	Macrophages	Activates TLR2-PI3K, blocks lysosomal fusion, and evades phagocytosis.	[187,188]
**Gingipains**	B cells	Upregulates CD19^+^ Breg levels to promote Treg hyperactivation; inhibits PI3K-Akt, reduces IL-6, and downregulates B10 cells (a Breg subset).	[185,186]
**Gingipains**	Oral–gut axis	Kills or degrades cytokines.	[149]
**Fimbriae**			
**FimA fimbriae**	Oral–gut axis	Activate TLR2/CXCR4, impair phagocytosis.	[181,182]
**Minor fimbriae**	DCs	Target DC-SIGN, evade dendritic cell autophagy and cytotoxic killing.	[179]
**Live** ***P. gingivalis***			
**Live** ***P. gingivalis***	TGF-β	Inhibits T cell activation; impairs antigen presentation.	[181]
**Live** ***P. gingivalis***	B cells	Promotes CD19^+^ Breg expansion.	[189,190]
**Live** ***P. gingivalis***	Erythrocyte	Binds erythrocytes and aberrantly activates complement receptor 3; induces immunosuppression.	[191]
**Other**			
**Supernatant**	T cells	Downregulates CD4/CD8 expression; inhibits T cell activation.	[162]
**OMVs**	DCs	Downregulate MHC-II, impair antigen presentation capacity.	[180]
**Capsule**	Neutrophils	Impairs NET recognition.	[19,183]
**Biofilms**	Neutrophils	Inhibit NET entrapment and recognition.	[183]

Abbreviations: CD19, cluster of differentiation 19; Breg, regulatory B cell; B10, IL-10-producing regulatory B cell subset; CXCR4, C-X-C motif chemokine receptor 4; DCs, dendritic cells; DC-SIGN, dendritic cell-specific intercellular adhesion molecule-3-grabbing non-integrin; MHC-II, major histocompatibility complex class II.

**Table 4:** Strength of evidence for mechanistic links between periodontitis and chronic liver diseases.

Mechanism	Highest Evidence Available	Other Supporting Data	Grade	Key Limitations
**Systemic inflammation**	Animal experiment [9,11,47,55,122]	In vitro study [113,114,118,119,120] Narrative review [15,16,17,21]	Moderate	No prospective cohort studies or randomized controlled trials; inflammatory mediators have not been quantified as causal intermediates.
**Hepatic cellular function**	Cross-sectional study [43]	Animal experiment [49,55,58,75,123,128,129,132,139] In vitro study [44,124,131,135,145]	Moderate	Limited primary hepatocyte evidence.
**Oral–gut–liver axis**	Cross-sectional study [111,153]	Animal experiment [9,128,151,152,154,157,159,161] In vitro study [158] Narrative review [55,56,60]	Moderate	No randomized controlled trials specifically targeting the oral–gut–liver axis.
**Ferroptosis**	Animal experiment [45,46,173,174]	In vitro study [44]	Moderate to Low	Highly dependent on a single research team; limited independent verification.
**Immune evasion**	Animal experiment [12,48,50]	Animal experiment [49,51,204] In vitro study [202,203,214]	Moderate	Difficult to distinguish from systemic inflammation.
**Endothelial-to-mesenchymal transition**	Indirect evidence from adjacent fields [52,53]	None	Low	Speculative mechanism: no direct evidence.

## Data Availability

No new data were created or analyzed in this study. Data sharing is not applicable to this article.

## Abbreviations

The following abbreviations are used in this manuscript:

*P. gingivalis*	*Porphyromonas gingivalis*
MASLD	Metabolic dysfunction-associated steatotic liver disease
EndMT	Endothelial–mesenchymal transition
CLDs	Chronic liver disease
*T. forsythia*	*Tannerella forsythia*
*F. nucleatum*	*Fusobacterium nucleatum*
LPS	Lipopolysaccharide
RANKL	Receptor activator of nuclear factor kappa-B ligand
TLR2	Toll-like receptor 2
TNF-α	Tumor necrosis factor-α
IL-1β	Interleukin-1β
IL-6	Interleukin-6
IL-12	Interleukin-12
OMVs	Outer membrane vesicles
MAPK	Mitogen-activated protein kinase
MMP-2	Matrix metalloproteinase-2
MMP-9	Matrix metalloproteinase-9
MMPs	Matrix metalloproteinases
CCL5	C-C motif chemokine ligand 5
CXCL8	C-X-C motif chemokine ligand 8
CXCL2	C-X-C motif chemokine ligand 2
MMP-1	Matrix metalloproteinase-1
IL-8	Interleukin-8
MCP-1	Monocyte chemoattractant protein 1
CCL3	C-C motif chemokine ligand 3
CX3CL1	C-X3-C motif chemokine ligand 1
RgpA	Arginine-specific gingipain A
RgpB	Arginine-specific gingipain B
Kgp	Lysine-specific gingipain
OPG	Osteoprotegerin
RANK	Receptor activator of nuclear factor kappa-B
NF-κB	Nuclear factor kappa-light-chain-enhancer of activated B cells
IFN-γ	Interferon gamma
PGE-2	Prostaglandin E2
RUNX2	Runt-related transcription factor 2
ALP	Alkaline phosphatase
ROS	Reactive oxygen species
As	Atherosclerosis
MASLD	Metabolic dysfunction-associated steatotic liver disease (updated nomenclature, formerly known as non-alcoholic fatty liver disease (NAFLD))
HCC	Hepatocellular carcinoma
MASH	Metabolic dysfunction-associated steatohepatitis
OR	Odds ratio
AST	Aspartate aminotransferase
ALT	Alanine aminotransferase
HBV	Hepatitis B virus
HCV	Hepatitis C virus
CHB	Chronic hepatitis B
CHC	Chronic hepatitis C
AIH	Autoimmune hepatitis
CAL	Clinical attachment loss
ALD	Alcohol-related liver disease
HR	Hazard ratio
TLRs	Toll-like receptors
TLR4	Toll-like receptor 4
MyD88	Myeloid differentiation primary response 88
ERK	Extracellular signal-regulated kinase
NLRP3	NOD-like receptor family pyrin domain containing 3
IL-17	Interleukin-17
NKs	Natural killer cells
DCs	Dendritic cells
NETs	Neutrophil extracellular traps
IFN-I	type I interferons
TLR9	Toll-like receptor 9
MEK/MKK	Mitogen-activated protein kinase kinase
Th17	T helper 17 cell
ERK1	Extracellular signal-regulated kinase 1
ERK2	Extracellular signal-regulated kinase 2
MPO	Myeloperoxidase
AIM2	Absent in melanoma 2
MDA	Malondialdehyde
STAT4	Signal transducer and activator of transcription 4
STAT5	Signal transducer and activator of transcription 5
PI3K	Phosphatidylinositol 3-kinase
Akt	Protein kinase B
HSCs	Hepatic stellate cells
LSECs	Liver sinusoidal endothelial cells
KCs	Kupffer cells
mtDNA	Mitochondrial DNA
ER	Endoplasmic reticulum
Bcl-2	B-cell lymphoma 2
Bax	Bcl-2-associated X protein
JNK	c-Jun N-terminal kinase
DISC	Death-inducing signaling complex
PERK	Protein kinase RNA–like endoplasmic reticulum kinase
eIF2α	Eukaryotic translation initiation factor 2 subunit alpha
LC3	Microtubule-associated protein 1 light chain 3
LAMP1/2	lysosomal membrane proteins 1/2
ASK1	Apoptosis signal-regulating kinase 1
MLKL	Mixed lineage kinase domain-like protein
GSDMD	Gasdermin D
TNFR	TNF-α-tumor necrosis factor receptor
MEKK	Mitogen-activated protein kinase kinase kinase
MKK4	Mitogen-activated protein kinase kinase 4
MKK7	Mitogen-activated protein kinase kinase 7
MKK3	Mitogen-activated protein kinase kinase 3
MKK6	Mitogen-activated protein kinase kinase 6
Rgp	Arg-gingipain
Kgp	Gingipain K
RIPK3	Receptor-interacting protein kinase 3
RIPK1	Receptor-interacting protein kinase 1
STAT3	Signal transducer and activator of transcription 3
PAR2	Protease-activated receptor 2
TGF-β1	transforming growth factor-β1
Smad2	SMAD family member 2
α-SMA	Alpha smooth muscle actin
ECM	Extracellular matrix
Gal-3	Galectin-3
Smad3	SMAD family member 3
Smad4	SMAD family member 4
OCLN	Occludin
RORγt	Retinoic acid receptor-related orphan receptor gamma t
DAMPs	Damage-associated molecular patterns
TLR5	Toll-like receptor 5
Treg	Regulatory T cell
TRIF	TIR-domain-containing adapter-inducing interferon-β
TSC1/2	Tuberous sclerosis complex 1/2
Rheb	Ras homolog enriched in brain
mTORC1	Mechanistic target of rapamycin complex 1
HMGB1	High mobility group box 1 protein
HSPs	Heat shock protein
NADPH	Nicotinamide adenine dinucleotide phosphate
GPX4	Glutathione peroxidase 4
ACSL4	Acyl-CoA synthetase long-chain family member 4
SLC7A11	Solute carrier family 7 member 11
MHC-II	Major histocompatibility complex class II
CXCR4	C-X-C chemokine receptor type 4
CD4^+^	Cluster of differentiation 4 positive
CD8^+^	Cluster of differentiation 4 positive
IL-10	Interleukin-10
CD19^+^	Breg CD19-positive regulatory B cell
TGF-β	Transforming growth factor-beta
PD-1	Programmed cell death protein 1
PD-L1	Programmed death-ligand 1
PECAM-1/CD31	Platelet endothelial cell adhesion molecule-1
FimA	Fimbriae protein A
IKKα	Inhibitor of κB kinase alpha
IKKβ	Inhibitor of κB kinase beta
IKKγ	Inhibitor of κB kinase gamma
IL-4	Interleukin-4
IL-18	Interleukin-18
IκB	Inhibitor of nuclear factor κB
p38	p38 mitogen-activated protein kinase
p50	Nuclear factor NF-κB p50 subunit
p65/c-Rel	Nuclear factor NF-κB p65 subunit/proto-oncogene c-Rel
Raf	RAF proto-oncogene serine/threonine-protein kinase
RAS-GTP	Ras-guanosine triphosphate
RCT	Randomized controlled trial
TGF-β1 receptor I	Transforming growth factor beta 1 receptor type I
TGF-β1 receptor II	Transforming growth factor beta 1 receptor type II
TLR3/4/7/9	Toll-like receptor 3/4/7/9
TNF-β	Tumor necrosis factor beta
Ub	Ubiquitin
VE-cadherin	Vascular endothelial cadherin
B10	IL-10-producing regulatory B cell subset
Breg	Regulatory B cell
C1q	Complement component 1q
C3	Complement component 3
C3a	Complement component 3a
C3b	Complement component 3b
C4	Complement component 4
C5	Complement component 5
C5a	Complement component 5a
C5b	Complement component 5b
DC-SIGN	Dendritic cell-specific intercellular adhesion molecule-3-grabbing non-integrin
